# Hormones and Cognition

**DOI:** 10.3390/brainsci11030318

**Published:** 2021-03-03

**Authors:** Caroline Gurvich, Natalie Thomas

**Affiliations:** 1 Monash Alfred Psychiatry Research Centre, Central Clinical School, Monash University and The Alfred Hospital, Melbourne, VIC 3004, Australia; natalie.thomas@unimelb.edu.au; 2 Department of Biochemistry and Pharmacology, Bio21, University of Melbourne, Parkville, VIC 3052, Australia

Neuroendocrine influences on cognitive (dys)function are well recognized. In particular, hormones related to the stress axis, the Hypothalamic–Pituitary–Adrenal (HPA) and the sex hormone axis, the Hypothalamic–Pituitary–Gonadal (HPG) are receiving growing attention in relation to their role in the development and manifestation of several psychiatric and neurological diseases that involve cognitive disruption [1]. For example, Alzheimer’s disease, depression, schizophrenia, and multiple sclerosis all have significant sex differences in terms of incidence and manifestation of symptoms [2]. When sex differences are observed, sex chromosomes and sex hormones are likely to be implicated [3]. Sex-steroid hormones that regulate reproductive function have multiple effects on the development, maintenance, and function of the brain, including significant effects on cognitive functioning [4]. Dysregulation of the stress hormone axis has been implicated as a key risk factor in virtually all psychiatric conditions, including schizophrenia, depression and complex trauma disorders. Research into how stress hormones and neuropeptides including cortisol, oxytocin, and vasopressin can moderate cognitive functioning is expanding [5]. The potential for developing and repurposing drugs that target hormones related to the HPA and HPG axes to treat cognitive problems is a growing and promising field; further research to clearly understand how stress and sex hormones affect the brain and behaviour across a lifespan is a necessary first step. This special issue sought to bring together research addressing the influence of stress and sex-steroid hormones on cognitive functioning and behaviour. 

The menstrual cycle is a model of hormonal flux that has garnered interest in understanding endogenous sex hormone influences on cognitive functioning and emotion processing. Premenstrual mood disorders, including premenstrual syndrome (PMS) and premenstrual dysphoric disorder (PMDD) have significant psychosocial impacts in the week(s) leading up to menstruation [6], but their neuropsychological profile is not well understood. In this special issue, Le et al. [7] provide a narrative review of studies investigating cognitive functioning in association with the menstrual cycle, with a focus on premenstrual mood disorders. Their review indicated that there are limited associations between cognition and the menstrual cycle phase in women without premenstrual mood disorders. In contrast, existing evidence, albeit limited, suggests that women with PMDD may experience cognitive disturbances premenstrually, particularly in the domain of executive function. The authors suggest that while endogenous sex hormones may influence cognitive processes over the menstrual cycle in both healthy and premenstrual disorder groups, the effects may only be clinically evident in the PMDD group.

Moving from endogenous to exogenous sex hormones, in this special issue Gurvich et al. [8] examine how the oral contraceptive pill influences cognitive functioning. Combined oral contraceptive formulations generally follow a 28-day cycle of 21–24 “active” pills containing synthetic analogues of estrogen and progesterone that provide negative feedback to the HPG axis, leading to a downregulation of endogenous sex hormone availability. Their study assessed cognitive performance in oral contraceptive users at different stages of the oral contraceptive cycle (active vs. inactive) and compared the performance among users of different oral contraceptive formulations according to their known androgenic activity. Their results suggested that users of oral contraceptive pills with androgenic activity demonstrate superior visuospatial ability and facial-affect discrimination tasks as compared to those who used formulations where the progestin had anti-androgenic properties. This study contributes to the growing understanding of the cognitive effects of oral contraceptive pills, particularly in relation to progestin androgenicity.

Sex-steroid hormones are thought to influence brain, behaviour and cognition via genomic and non-genomic actions although a complete understanding of the mechanisms by which sex-steroid hormones influence cognition is lacking. One potential mode of action could involve cholinergic neurotransmission, and this is explored in this special issue by Ch’ing et al. [9]. Their study investigated how a lack of ovarian hormones following an ovariectomy may have an impact on muscarinic receptor-induced deficits in prepulse inhibition (PPI, a measure of sensorimotor gating) and muscarinic receptor density in several brain regions in adult female rats. Their results did not observe any ovariectomy effects on scopolamine-induced impairments in PPI or any significant changes in ovariectomized rats relative to intact rats in the binding density of muscarinic receptors in brain regions relevant to cognition. These results suggest that removing peripheral ovarian hormones does not influence the cholinergic muscarinic receptor system in the context of PPI or receptor binding density. Further exploration of potential pathways that ovarian hormones may have effects on cognition, other than the cholinergic muscarinic receptor system, is encouraged.

This special issue also explores stress hormone influences on cognition. Two articles are included that examine work stress [10] and stress in the context of dual tasking [11]. Dumant et al. [10] explored whether sex differences in memory biases towards work-related stress information may explain previously reported sex differences in work-related stress [12]. Using their novel Work-Stress Memory Task (WSMT), developed to assess recall of information related to (1) stress at work, (2) positive information, or (3) neutral information, Dumant et al failed to find sex differences in relation to work-stress memory biases; however, women recalled more positive and neutral words than men, suggesting a sex difference in the processing and recalling of non-threatening information. The results did not demonstrate associations between memory performance and either reactive or diurnal cortisol for either males or females. Hence, findings from this study suggest that sex-specific cognitive biases may not be an explanatory factor for sex differences in stress-related mental health disorders in healthy male and female workers.

In an older adult population, Condello et al. [11] investigated stress responses and perceived effort during walking exercises with and without a concurrent working memory task. Results indicated locomotor-cognitive dual-tasking elicited a higher perceived effort and higher autonomic stress response (using salivary α-amylase) compared to the locomotor or the cognitive task performed on its own, that is, single-task. The study was also interested in whether regular physical activity might buffer the autonomic response to acute stressors in physical and cognitive domains; however, the results did not support this cross-stressor adaptation hypothesis. Their results did suggest that participants who engaged in regular physical activity exhibited an association between their autonomic stress response during dual-tasking and their perceived exertion and overt performance, suggesting regular physical activity may assist older adults to balance stress and align effort to match task demands.

We hope that the topics addressed in this special issue will continue to stimulate research into the neuroendocrinology of cognition, particularly the role of stress and sex hormones.
